# T-Wave Inversion After Escitalopram Overdose: A Case Report

**DOI:** 10.7759/cureus.34523

**Published:** 2023-02-01

**Authors:** Preethy Koshy, Gajanan Chavan, Govind Nagdev

**Affiliations:** 1 Emergency Medicine and Critical Care, Jawaharlal Nehru Medical College, Datta Meghe Institute of Medical Science, Wardha, IND; 2 Emergency Medicine, Jawaharlal Nehru Medical College, Datta Meghe Institute of Medical Science, Wardha, IND

**Keywords:** ssri, qtc prolongation, t wave inversions, ecg changes, overdose, emergency medicine

## Abstract

Selective serotonin reuptake inhibitors (SSRIs) are the most common antidepressants used due to their comparatively less cardiotoxic effects than tricyclic antidepressants. Corrected QT interval (QTc) prolongation is the most common electrocardiography (ECG) change that has been encountered with SSRI overdose. This case report is about a 22-year-old woman who was brought to the emergency department (ED) with an alleged history of consumption of 200 mg of escitalopram. Her ECG showed T-wave inversions in anterior leads one to five, which reverted (in leads four and five) the next day with supportive management. After 24 hours, she developed dystonia, which resolved with mild doses of benzodiazepine. Hence, ECG changes like T-wave inversions may occur even with a small overdose of an SSRI without any significant adverse effects.

## Introduction

Drug-induced arrhythmias can lead to serious cardiovascular events such as torsade de pointes and sudden cardiac arrest [1]. QT interval prolongation is accepted as a surrogate marker for identifying the proarrhythmic potential of a drug under development [2]. Various anti-depressants and antipsychotic medications have been shown to cause prolongation of the QT interval in adults across a wide age range [3]. The cardiovascular effects of antidepressants have been the subject of recent debate, and the dose of citalopram was modified owing to the risk of QTc prolongation [4].

Escitalopram, which is a selective serotonin reuptake inhibitor (SSRI), is an S-enantiomer of citalopram used for the treatment of anxiety disorder and depression. According to FDA reports, thorough QT (TQT) studies showed that citalopram causes significant prolongation of the QT interval at a dose of 60 mg/day, whereas escitalopram causes a prolongation of the QT interval in a dose-dependent manner without clinically relevant findings [5]. However, there are no studies showing that QTc prolongation can have clinically negative outcomes, such as sudden cardiac death [6].

This case report is about a 22-year-old woman who presented to the emergency department (ED) with an escitalopram overdose and developed ECG changes.

## Case presentation

A 22-year-old woman was brought to the emergency department with a history of consuming 20 tablets of escitalopram (10 mg) one hour prior to her presentation to the ED, followed by one episode of vomiting and agitation after that. She had a medical history of depressive disorder for two years, during which she was on escitalopram and clonazepam tablets.

On physical examination, her vitals were as follows: pulse rate: 102/min, blood pressure: 130/80 mm Hg, respiratory rate: 20/min, oxygen saturation: 97% on room air, and temperature: 97.6°F. There were old hesitation cuts on the flexor aspect of her left wrist. She was fully conscious and oriented, and her systemic exam was within normal limits.

**Figure 1:**
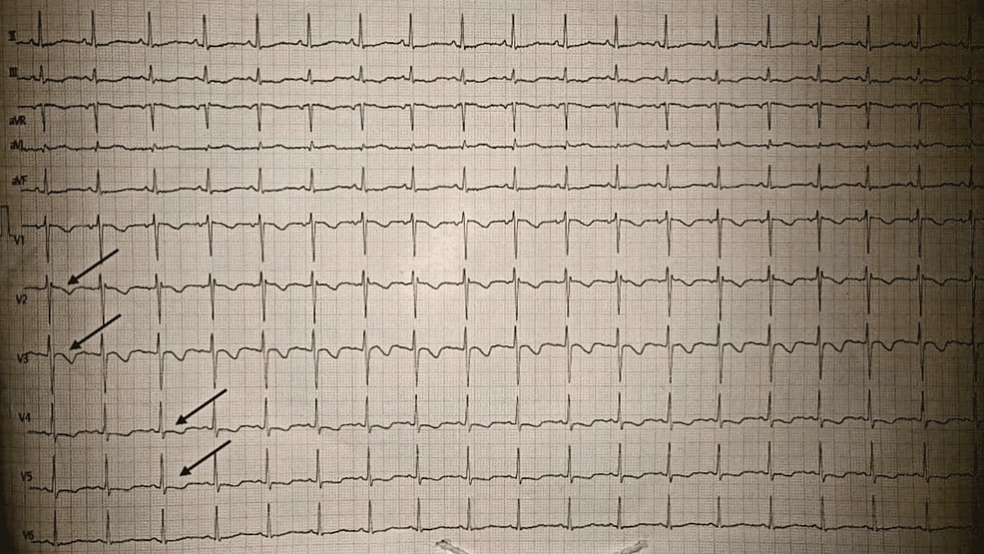
An ECG showing T-wave inversions in precordial leads

An ECG demonstrated T-wave inversions in the precordial chest that led from one to five, as can be seen in Figure 1.

The patient was given gastric lavage in the emergency department, and her vitals were closely monitored. Routine investigations, comprising a complete hemogram, liver function tests, and kidney function tests, were within normal limits. A gastric lavage sample sent for toxicologic analysis confirmed the diagnosis.

**Figure 2:**
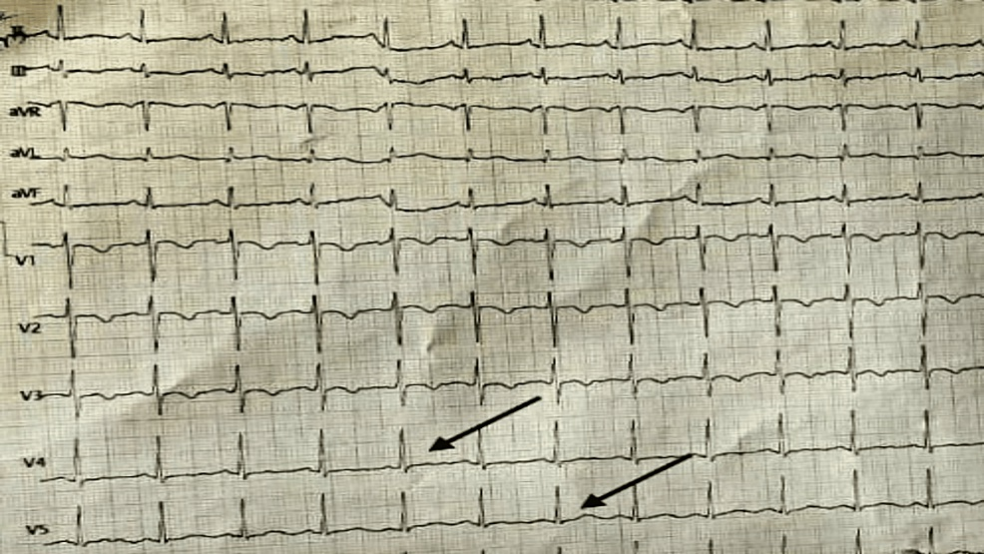
A repeat ECG showing upright T-waves in precordial leads four and five

The patient was admitted to the intensive care unit. The two-dimensional echo showed a normal study with good left ventricular systolic function (LVEF of 60%). She was given psychotherapy in the form of dialectical behavior therapy, which consisted of group skills training, individual therapy, etc. Her ECG changes reverted in leads four and five the next day, as can be seen in Figure 2.

Twenty-four hours later, the patient developed dystonia and exaggerated bilateral knee jerks. Magnetic resonance imaging of the brain showed no obvious abnormalities in the brain parenchyma. She was managed with low doses of benzodiazepines and her symptoms subsided gradually. She became stable and was discharged on the fifth day after admission.

## Discussion

Overdoses with SSRIs alone rarely cause fatalities, and most patients improve without any sequelae. A moderate overdose (30 times the daily dose) leaves no or minor symptoms. At 50-75 times the daily dose, there can be symptoms like drowsiness, tremor, nausea, and vomiting. At higher doses, serious adverse effects like ECG changes, decreased level of consciousness, and seizures may occur. Fatalities may occur with exceptionally large acute overdoses (>150 times the usual daily dose), especially when co-ingested with other drugs [7].

A comparative analysis of antidepressant overdoses in suicidal adults found that although all SSRIs had low hazard indices, citalopram had the highest hazard index among the SSRIs [8]. In addition to the mild transient complaints seen with all SSRIs, citalopram produces seizures and QT prolongation, which ranges from 12% to 68% in case series involving admitted adult patients [9]. Patients have developed torsades de pointes (TdP) with either therapeutic or supratherapeutic doses of citalopram [10-13].

This case report is of a 22-year-old woman who presented to the emergency department with an SSRI overdose of 200 mg and symptoms of irritability and agitation. Her ECG showed T-wave inversions in the anterior chest leads (one to five), which reverted after supportive management without any two-dimensional echo changes or adverse clinical sequelae. She developed dystonia, which gradually subsided with benzodiazepines.

ECG changes with such a small overdose of SSRI have not been reported in the literature to date. The majority of the studies reported showed QTc prolongation as ECG changes with SSRI overdosage, but no studies showing T-wave inversions were reported. However, other physiological causes of T wave inversions, such as those seen in anemic patients, females, and athletes, should be considered. Although very little is known about the adverse effects of SSRI overdose, very few case fatalities have been reported, making them safer than tricyclic antidepressants (TCAs). More research should be done on patients focusing on ECG changes with mild SSRI overdoses to see if there is a causal relationship. 

## Conclusions

T-wave inversion with SSRI overdose has not been reported to date. ECG changes may occur with a mild overdose of an SSRI. Hence, close monitoring is recommended in patients with SSRI overdose, even though SSRIs are considered much safer than tricyclic antidepressants. T-wave inversions may be seen in the anterior leads in normal individuals also, which may produce a dilemma in diagnosis. However, a thorough history-taking and investigation may aid in the diagnosis.
